# Perceived health facility-related barriers and post-abortion care-seeking intention among women of reproductive age in Osun state, Nigeria

**DOI:** 10.1186/s12905-023-02464-3

**Published:** 2023-06-16

**Authors:** Tosin Olajide Oni, Stephen Ayo Adebowale, Anuoluwapo Adeyimika Afolabi, Akanni Ibukun Akinyemi, Olufunmilayo Olufunmilola Banjo

**Affiliations:** 1 Department of Demography and Social Statistics, Obafemi Awolowo University, Ile-Ife, Nigeria; 2 Department of Epidemiology and Medical Statistics, Faculty of Public Health, University of Ibadan, Ibadan, Nigeria; 3 Population and Health Research Entity, North-West University, Mafikeng, South Africa; 4 Department of Community Medicine, Faculty of Public Health, College of Medicine, University of Ibadan, Ibadan, Nigeria

**Keywords:** Post-abortion care, Health facility barriers, Osun state, Care-seeking intention, Nigeria

## Abstract

**Background:**

Post-abortion care (PAC) prevents death and complications caused by unsafe abortion which is widespread in Nigeria. Yet, there is sparse community-based evidence on women’s intention to seek PAC should they have an abortion. This study examined the influence of perceived health facility-related barriers (HFRB) on post-abortion care-seeking intention (PACSI) among women of reproductive ages in Osun state, Nigeria.

**Methods:**

The study focused on women in a sexual relationship and who were residents of Osun state. A community-based survey was implemented using a multi-stage sampling technique. The calculated sample size (with attrition) was 1200 and data were collected from women aged 15-49 years, using open data kit (ODK). However, 1,065 complete responses were received on the ODK server, indicating an 88.8% response rate. Models were estimated using ordered logistic regression (Ologit) (α_0.05_) and data analysis was performed using Stata 14.0.

**Results:**

Mean age of the women was 29.3±7.6 years and 34.01% had the intention to seek PAC in health facilities. Lack of service confidentiality and unavailability of equipment specific to abortion were the two most reported barriers that would prevent women from seeking PAC. The adjusted Ologit model showed that respondents with perceived low HFRB had higher odds (aOR=1.60; CI=1.12-2.11) of seeking PAC in the health facility. Also, women who were employed and skilled were more likely (aOR=1.51; CI=1.13-2.01) while women who had PAC support from spouses/partners had higher odds of healthy PACSI (aOR=2.03; CI=1.48-2.78). Other identified predictors of PAC seeking intention included level of education, employment status, and spousal/partner support.

**Conclusion:**

Perceived lack of trust in service provision and necessary equipment specific to abortion care had a negative influence on women’s PACSI in Osun state. Reassuring health interventions that focus on improving the public perception of healthcare services and confidence to use the facility will likely improve the patronage of health facility for post-abortion care in Osun sate.

## Background

Post-abortion care (PAC) has been used worldwide to save women’s lives. It prevents/reduces the severity of complications following an abortion [1, 2]. PAC refers to the bundle of care that is offered to women after undergoing any form of abortion, whether induced or spontaneous. The World Health Organization [3] notes that ensuring that women and girls have access to PAC is fundamental to meeting the Sustainable Development Goals (SDGs) relating to good health and well-being (SDG3) and gender equality (SDG5). PAC is helpful, not only in preventing death and in treating complications from abortion (both safe and unsafe), but also in providing a platform through which women are educated on the need for comprehensive family planning to prevent a reoccurrence [4].

Unlike abortion which is legally restrictive and shrouded in socio-religious stigma across the world [5, 6], PAC is not illegal and women who seek PAC should be less denied by providers in many countries. However, it appears that in developing countries, many women have written off the need for PAC, perhaps due to a lack of information about the legality of PAC or some perceived health-facility-related barriers (HFRB). Some commonly reported HFRBs are lack of service confidentiality, poor satisfaction, service delay, and high costs of service amongst others [7]. In Nigeria for instance, no less than 1.25 million abortions occur annually [8, 9] and the legal restriction and social stigma surrounding abortion in the country [9], which keep people away from facility abortion, make majority of the abortions potentially unsafe. Yet, only 5.6 per 1000 women received PAC in health facilities in Nigeria [8]. Given the low PAC in the face of high level of unsafe abortion in Nigeria, the country is arguably a significant contributor to the 185 deaths per 100,000 abortions in sub-Saharan Africa in 2020 [10].

Much of the efforts to address unsafe abortions in Nigeria have focused on preventing unwanted pregnancies through widespread contraceptives. The effectiveness of modern contraceptives is in no doubt and has been well-published [11, 12]. However, data from the Nigeria Demographic and Health Survey (NDHS) show that despite the reported high usage of contraceptives in southern Nigeria (24.9% in 2013 and 24.3% in 2018) compared with northern Nigeria (12.4% in 2013 and 10.9% in 2018) [13], unwanted pregnancies were much higher in the South (24.7%) than in the North (9.9%). Osun State with the prevalence of unwanted pregnancy of 17.1% was the most hit among the south-western states while Ogun state was the least (12.8%). Osun is one of the states with high prevalence of unwanted pregnancy across the 36 states in Nigeria. Akwa Ibom state has the highest prevalence (28.5%), followed by Cross Rivers state (20.6%), and the least was observed in Borno state (2.5%) [13]. Given the reports that up to 60% [3] of unintended pregnancies are resolved through abortion, several unsafe abortions must have occurred and may continue to occur in Osun state, given the legal restrictions on safe abortion in Nigeria. PAC then becomes extremely important to saving women’s lives and for preventing/reducing abortion-related disabilities among women who may have an abortion in the State.

Studies have recommended PAC service provision in Nigeria [14, 15]. However, for PAC service provision to be effective, it is essential to understand perceived barriers that could inhibit PAC-seeking intention (PACSI) among women, given the sensitive nature of abortion-related services. PACSI refers to women’s readiness to seek PAC services in a health facility capable of rendering the services immediately following an abortion [3]. Even though PACSI does not represent a real behavior, the theory of planned behavior (TPB) posits that behavioral intentions are the precursors and proximate determinants of actual performance [16]. Several studies have investigated behavioral intentions as a way of understanding potentially dangerous behavior to determine a potential need for health-promoting interventions aimed at inducing positive behavioral change [17–20].

Despite the overwhelming evidence of the high rate of unsafe abortion in Nigeria, no known study has investigated women’s PACSI. The majority of studies conducted on PAC in Nigeria have mainly been facility-based [21, 22]. Such studies must have grossly over-reported PAC-related indices because it was estimated that for every 1 woman who receives PAC in a Nigerian facility, almost 4 are not seen [8]. Plausibly though, the aforementioned PAC studies were made facility-based because of the sensitivity and potential stigma that could make women deliberately distort their abortion experience in a community-based survey, for the sake of social desirability. In this study, PACSI was investigated as a proximate indicator of the PAC situation in Osun state, and deliberate distortion was not envisaged because no reference was made to individuals’ abortion experience.

## Methods

### Study design and participants

A cross-sectional design was used to provide a snapshot of women’s PACSI and the associated factors in Osun state - one of the six states in South-west Nigeria. The study participants were women of reproductive ages who were in sexual relationships, irrespective of marital status or residential arrangement with spouses or sexual partners. A community survey was conducted in which quantitative data were collected through the use of structured questionnaires. Questionnaire contents were scripted into Open Data Kit which was used for electronic data collection by ten (10) trained female Research Assistants (RAs). The data collection instrument was tested in a pilot study conducted among 120 women (10% of the calculated sample size). This was done in November 2022 in Ibadan, Oyo state. The reliability analysis of the instrument showed a Cronbach’s Alpha coefficient of 0.823. The data collection process took 12 days in December 2022.

### Sample and sampling

The sample size was calculated as 1150 using Leslie Kish’s formula for calculating sample size in descriptive studies [23]. The formula is applicable for studies in which the proportion (p) or the best guess about the proportion of interest is known. It was reported as 0.523 [21] and the statistical table value (Z_0.05_) is 1.96 at 0.025 tolerance level (d).

Multi-stage sampling was done with the aid of the Osun state map designed by the National Population Commission (NPC). The map illustrates the spatial distribution of LGAs and political wards within the state and this was used to stratify the LGAs and the wards into non-overlapping subsets. Sampling stage one: the 30 LGAs were stratified into three homogenous groups (senatorial districts), out of which two LGAs were selected from each of the strata, making a total selection of six LGAs. Sampling stage two: Since the LGAs have clearly defined enumeration areas (by the NPC) with clear and identifiable boundaries, 4 enumeration areas (2 from each rural and urban) were selected from the list of political wards in each LGA, thereby making a total selection of 24 enumeration areas in the six LGAs.

Stage Three: Here, a listing of housing units within each selected enumeration area was conducted and a sampling frame of all the eligible respondents within each housing unit was prepared. In the subsequent stages, a systematic random sampling technique was used to select the eligible respondents from the sampling frame. In all, at least 50 eligible respondents were targeted from each EA, thus potentially recruiting 24*50 (1200) respondents across the state. However, a total of 1065 complete quantitative responses were received in the ODK server at the end of data collection.

### Measures

The outcome variable is women’s post-abortion care-seeking intention (PACSI). It refers to women’s intention and readiness to access care in a healthcare facility should they have an abortion. It was measured from two primary questions which each had a follow-up question. The first primary question was ‘should women seek -post-abortion care?’, followed by ‘where should women seek -post-abortion care?’. This question was asked to examine the perceptive dimension to behavioural intention, which is a proximate determinant of real behaviour, in consonance with the postulation of the theory of planned behaviour. The second primary question was ‘would you seek post-abortion care?’, followed by ‘where would you seek post abortion?’. Those who responded *yes* (1) to the primary questions were asked the follow-up questions while those who responded *no* (0) were not asked. The follow-up questions permitted multiple responses but only the choice of *skilled health workers* was reckoned with in the computation of the outcome variable. Respondents who chose *skilled health workers* in the two follow-up questions scored ‘2’ and were labeled as those with *healthy* PACSI. Those who chose *skilled health workers* only in either of the two follow-up questions scored ‘1’ and were labeled ‘*baseline*’ while all the respondents who responded ‘no’ to any of the primary questions scored ‘0” and were labeled as *unhealthy.* The place dimension in PAC was added because it has to be a health facility to be considered safe.

The principal explanatory variable is the health facility-related barriers. These refer to the respondents’ perception of six commonly reported barriers to healthcare services [7] which could potentially impair health-seeking behavior, such as seeking post-abortion care services in health facilities. These are: (i) unaffordability (cost) of services (ii) lack of confidentiality (iii) service delay (iv) poor attitude of healthcare workers (v) non-availability of equipment specific to abortion care, and (v) low service satisfaction. The respondents were asked, on a *yes*(1) or *no*(0) response, if any of these could prevent them from accessing PAC. The response scores were composited and then disaggregated into three groups using the scores’ mean (x̄) and the standard deviation (SD), i.e. [x̄±SD]. The groups were *low-level barriers* (minimum score to [<x̄ - SD]), *mid-level barriers* ([x̄-SD] to [x̄+SD]), and *high-level barriers* (> x̄+SD to maximum score).

The variable selection for the explanatory factors was strictly informed by the TPB framework [16] and its adaptation in previous studies [17, 19, 20]. The TPB’s control factors are the external and internal factors that may inhibit or facilitate the behavioral intention. One of these is the facility-related barriers. Others are transportation barriers and distance from the facility. The TPB’s motivation to comply is conceptualized as women’s involvement in household decision-making, partner/spousal support, opinion (positive) of significant others, and the women’s socio-demographic characteristics. Partner/Spousal support was measured as the participants’ perception of whether their partners/spouses would permit them to access PAC, provide them with finance, accompany them, and provide them with moral support. The responses to the variables were aggregated into *available* where the partners/spouses would do at least 2 of the 4 aforementioned or *not available* where partner/spousal support covered less than two. Participants who could decide on their healthcare, how they spend their earnings, large household purchases, visits to family/relatives, and on what spouses/partners earned were categorized as ‘autonomous household decision makers’. Those who would have to do this in conjunction with their partners/spouses were grouped as ‘intermediate decision makers’ while those who had no say in these decisions at all were grouped as ‘dependent’ household members in decision-making. The participants’ significant others were regarded as friends, religious leaders, relatives, and opinion leaders. How the opinions (*positive* or *negative*) of these individuals about PAC influence the participants’ intention was also examined.

The participants’ socio-demographic characteristics were age, marital status, the highest level of education, employment status, type of place of residence, and frequency of exposure to the media. Participants’ exposure to the media was measured using the reported frequencies of reading newspapers, listening to the radio, and watching television. The responses were *not at all*, *less than once a week*, *once a week,* and *almost every day*. The four variables were aggregated and the responses were used in computing composite scores that were grouped into *always* (almost every day), *often* (once a week), *rarely* (less than once a week), and *never* (not at all).

### Data analysis

Frequency and percentage distributions were used for describing all the background categorical variables (e.g. educational attainment) and mean analysis was used for presenting the numeric background characteristics (age as at previous birthday and average monthly income). Before fitting the models, a test of multicollinearity was performed to detect collinear variables that could affect how reliable the regression slopes would be. In this, a variance inflation factor (VIF) analysis was performed. The results showed that there was no serious multicollinearity among the explanatory variables, given that each variable had less than a VIF score of 5. A VIF of 5 is the rule of thumb, indicating that the concerned variables are not multicollinear [24].

Ordered logistic regression (OLogit) models were fitted to estimate effects on PACSI. Model 1 was fitted at the bivariate level of analysis to estimate the crude main effect of the main explanatory variable (health service-related barriers) as well as each of the other explanatory factors on PACSI. The Table presenting the bivariate models also shows how the estimated prevalence of healthy PACSI changes across the levels of each independent variable. At the multivariate level, three models were fitted. The first multivariate model (model 2) estimated the influence of all the socio-demographic variables on PACSI. Model 3 estimated the influence of all other explanatory variables on PACSI. The final model contained all the explanatory variables and estimated their effect on PACSI. This final model formed the basis of the conclusion of the study.

Fitting OLogit models is justifiable when estimating the effects of factors on a categorical outcome variable in which the levels are more than two and have a meaningful order [23, 25]. In this study, the application of OLogit is justified because the outcome variables met these requirements. Stated specifically, the three levels of the outcome are healthy (2), intermediate (1), and unhealthy (0). The odds ratio (OR) of the OLogit models was used to interpret and explain the influence of the factors while their statistical significance was tested at a 5% level of significance and 95% confidence interval. Stata 14 [26] was used for the data analysis.

### Ethical considerations

The Health and Research Ethics Committee of the Institute of Public Health, Obafemi Awolowo University reviewed and approved the study protocol and data collection instrument with approval code IPH/OAU/12/18/53. Participation was purely voluntary and premised on informed consent.

## Results

**Fig. 1:**
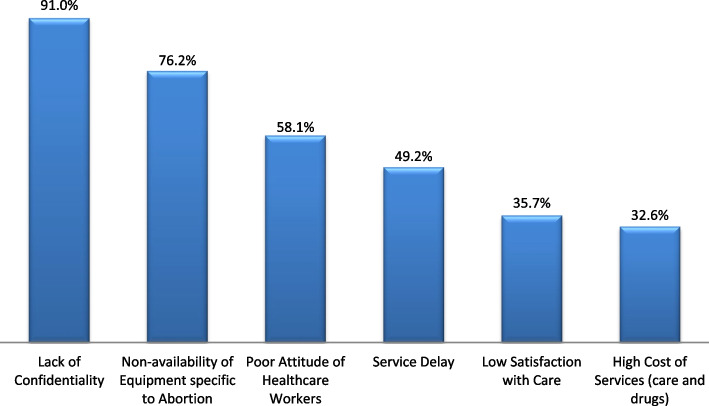
Health facility-related barriers that would prevent women from accessing PAC

Table 1 presents results on the socio-demographic characteristics of the women used as respondents for the study. The results show that the highest proportion (43.5%) of the respondents were aged 25-34 years while the lowest proportion (4.5%) were aged below 18. The mean age was 29.3±7.6 years. While 45.5% had secondary education, 3% had no completed level of education and 53.4% were married/cohabiting. The majority of the respondents practiced Christianity (44.4%) and the employment status showed that 45.6% were skilled employees.Results on the type of place of residence show that 57.4% and 42.6% of the respondents lived in urban and rural communities respectively. The results also show that the highest proportion (42.2 %) of the respondents were dependent when it had to do with making household decisions. While 40.7% jointly made decisions with their spouses/partners, 17.1% reported that they had the autonomy to make decisions in their household. Results as illustrated in Fig. 1 show that lack of service confidentiality was the most commonly perceived health facility-related barrier as it was reported by 91%. This was followed by the non-availability of equipment specific to abortion which was perceived as a barrier by 76.2%. Two barriers least perceived by the respondents were the high cost of services (32.6%) and low satisfaction with care provision (35.7%).

**Table 1:** Respondents’ socio-demographic characteristics

**Variables**	**Categories**	**Frequency (*N*=1062)**	**Percentage (%)**
Participants’ Age (*in years*) Mean Age = 29.3±7.6	Below 18	48	4.5
	18-24	274	25.8
	25-34	462	43.5
	35-49	278	262.
Highest Level of Completed Education	None	27	2.5
	Primary	125	11.8
	Secondary	483	45.5
	Tertiary	427	40.2
Marital Status	Never married	308	29.0
	Married/Cohabiting	567	53.4
	Others	187	17.6
Religion	Christianity	472	44.4
	Islam	438	41.2
	Others	152	14.3
Employment Status	Unemployed	315	29.7
	Employed: unskilled	263	24.8
	Employed: skilled	484	45.6
Type of Place of Residence	Rural	452	42.6
	Urban	610	57.4
Frequency of Exposure to the Media	Never	208	19.6
	Rarely	351	33.1
	Often	382	36.0
	Always	121	11.4
Involvement in Household Decision Making	Dependent	448	42.2
	Intermediate	432	40.7
	Autonomous	182	17.1

### Results from the bivariate models

Results as presented in Table 2 (Model 1) show that health service-related barriers had a significant effect on women’s PACSI as the odds of having a healthy PACSI increased significantly with lower levels of barriers. Stated empirically, women who experienced low-level barriers had 55% (OR=1.55, CI: 1.19 2.01) significantly higher odds than women who had high-level barriers to having healthy PACSI. Also, women who had mid-level barriers had 35% (OR=1.35, CI: 1.02 1.80) higher odds of having a healthy intention about PAC. Furthermore, the results indicate that even though the odds of having a healthy intention about PAC increased with participants’ ages, the odds were not statistically significant. This was the same as the odds of a healthy PACSI across the respondents’ marital status, religion, residence, and women’s decision-making power in the household. However, employment status had a significant influence on PACSI. In this, women employed in skilled occupations were 48% (OR=1.48, CI: 1.14 1.92) more likely than unemployed women to have a healthy PACSI. Also, women who were always exposed to the media had a 54% (OR=1.54, CI: 1.02 2.31) higher likelihood, than women who were never exposed to the media, of having a healthy PACSI. Women who would enjoy spouses/partners’ support for PAC also had 61% (OR=1.61, CI: 1.26 2.07) significantly higher odds of healthy PACSI, than women who reportedly would not have their spouses/partners’ support for PAC.

**Table 2:** Percent prevalence of healthy PACSI and bivariate relationships

PACSI	Healthy (34.09%)					
	Baseline (28.34%)					
	Unhealthy (37.57)	% healthy PACSI	Model 1			
			OR	95% CI	OR	95% CI
Health service-related barriers	High	23.55	RC			
	Medium	36.92	1.35^a^	1.02 1.80		
	Low	41.10	1.55^a^	1.19 2.01		
Maternal Age	Pre	27.08			RC	
	Early	31.75			1.09	0.63 1.91
	Mid	38.74			1.37	0.80 2.35
	Advanced	29.86			1.12	0.64 1.95
Highest Level of Education	None	37.04			RC	
	Primary	24.00			0.52	0.25 1.10
	Secondary	35.82			0.81	0.41 1.63
	Tertiary	34.89			0.72	0.36 1.44
Marital Status	Never	30.52			RC	
	Married/C	36.16			1.19	0.92 1.54
	Others	33.69			1.16	0.83 1.62
Religion	Christianity	35.38			RC	
	Islam	31.96			0.96	0.75 1.22
	Others	36.18			1.24	0.89 1.73
Employment Status	Unemployed	28.25			RC	
	Employed: unskilled	35.36			1.30	0.96 1.75
	Employed: skilled	37.19			1.48^a^	1.14 1.92
Residence	Rural	32.08			RC	
	Urban	35.57			1.14	0.91 1.42
Frequency of Media Exposure	Never	25.00			RC	
	Rare	38.75			1.20	0.88 1.63
	Often	32.20			1.03	0.76 1.40
	Always	42.15			1.54^a^	1.02 2.31
Household Decision Power	Dependent	33.26			RC	
	Intermediate	36.81			0.90	0.71 1.16
	Autonomous	29.67			0.95	0.70 1.30
Support from Spouse/Partner	Not Available	29.68			RC	
	Available	45.07			1.61^a^	1.26 2.07
Transportation & Distance	Not a barrier				RC	
	It is a barrier				0.99	0.78 1.26
Opinion of Significant Others	Negative	33.53			RC	
	Positive	36.50			1.07	0.80 1.42

*OR* Odds Ratio, *CI* Confidence Interval, *RC* Reference Category

^a^significant at 5% level

### Results from multivariate models

Model 2 (in Table 3) shows that the influence of health service-related factors on PACSI increased when the respondents’ socio-demographic characteristics were adjusted for. Stated specifically, the odds of a healthy PACSI increased from 55% when no variable was controlled for, to 58% (aOR=1.58, CI: 1.20 2.07) when the socio-demographic characteristics were adjusted for. At this level, respondents who had mid-level barriers no longer had a significant likelihood of a healthy PACSI. Also on the adjusted model, skilled employment became significant in predicting women’s PACSI, unlike the non-significant odds of women’s employment status on the unadjusted model. Here, skillfully employed women had 54% (aOR=1.54, CI: 1.16 2.05) higher likelihood than unemployed women to have a healthy PACSI. In model 3, health service-related barriers retained their significant influence on women’s PACSI. Here, women who had low-level health service-related barriers were 56% (aOR=1.56, CI: 1.20 2.04) more likely, than women with high-level barriers, to have a healthy PACSI. Also, women whose spouses/partners would support PAC had a 91% (aOR=1.91, CI: 1.41 2.59) higher likelihood of a healthy PACSI than women whose spouses/partners would not support PAC.

**Table 3:** Influence of perceived health facility-related barriers on PACSI

		Model 2		Model 3		Model 4	
		aOR	95% CI	aOR	95% CI	aOR	95% CI
Health service-related barriers	High						
	Medium	1.34	0.99 1.82	1.29	0.93 1.80	1.23	0.87 1.75
	Low	1.58^a^	1.20 2.07	1.56^a^	1.20 2.04	1.60^a^	1.22 2.11
Maternal Age	Pre						
	Early	0.98	0.55 1.76			1.05	0.58 1.89
	Mid	1.14	0.60 2.16			1.28	0.67 2.45
	Advanced	0.85	0.43 1.69			1.00	0.50 1.99
Highest Level of Education	None						
	Primary	0.45^a^	0.21 0.98			0.37^a^	0.17 0.81
	Secondary	0.67	0.32 1.40			0.60	0.28 1.26
	Tertiary	0.53	0.25 1.14			0.49	0.23 1.07
Marital Status	Never						
	Married/C	1.04	0.73 1.48			1.03	0.72 1.47
	Others	1.17	0.73 1.88			1.13	0.70 1.82
Religion	Christianity						
	Islam	0.94	0.73 1.21			0.89	0.68 1.15
	Others	1.25	0.88 1.78			1.26	0.88 1.80
Employment Status	Unemployed						
	Employed: unskilled	1.27	0.93 1.74			1.36	0.98 1.87
	Employed: skilled	1.54^a^	1.16 2.05			1.51^a^	1.13 2.01
Residence	Rural						
	Urban	1.16	0.91 1.46			1.18	0.93 1.50
Frequency of Media Exposure	Never						
	Rare	1.13	0.81 1.58			1.10	0.79 1.54
	Often	0.92	0.66 1.27			0.85	0.61 1.19
	Always	1.32	0.85 2.04			1.20	0.77 1.87
Household Decision Power	Dependent						
	Intermediate			0.86	0.67 1.10	0.78	0.60 1.01
	Autonomous			0.90	0.66 1.23	0.91	0.66 1.26
Support from Spouse/Partner	Not Available						
	Available			1.91^a^	1.41 2.59	2.03^a^	1.48 2.78
Transportation & Distance	Not a barrier						
	It is a barrier			0.80	0.60 1.06	0.84	0.63 1.13
Opinion of Significant Others	Negative						
	Positive			0.80	0.57 1.13	0.78	0.55 1.11

*OR* Odds Ratio, *aOR* Adjusted Odds Ratio, *CI* Confidence Interval, *RC* Reference Category

^a^significant at 5% level

The final model (model 4) shows that the significant likelihood of having a healthy PACSI was at the highest when the women’s socio-demographic characteristics and other variables were adjusted for. In other words, if the differences in the women’s socio-demographic characteristics, support from spouses/partners, and opinions of significant others were held constant, those who had low-level health facility-related barriers were 60% (aOR=1.60, CI: 1.22 2.11) more likely to have a healthy PACSI than women who had high-level barriers. Having skilled employment also maintained its significant influence on PACSI as skillfully employed women had 51% (aOR=1.51, CI: 1.13 2.01) higher likelihood of having a healthy PACSI, than unemployed women. In addition, spousal/partner’s support maintained its significant influence on PACSI as women who would be supported by spouses/partners were more than twice likely (aOR=2.03, CI:1.48 2.78) to have a healthy PACSI than women who reported that their spouses/partners would not support them for PAC.

## Discussion

This study was a community-based survey of women in sexual relationships. The study identified some underlying health facility-related barriers that could make abortion continue to be a significant source of maternal death and disability in Osun state. Findings revealed a 34.1% prevalence of healthy PACSI in the state. This result, however, was lower than PAC demand reported in an earlier study [21]. A plausible reason that could account for the difference in the prevalence found in this study and those reported by other studies is that while most of the previous studies examined real PAC-seeking behavior and were mostly facility-based, this study was community-based and examined behavioral intention. Except for women who had a spontaneous abortion or those who had an abortion to save their life (when the fetus becomes a threat to the carrier’s life), women who had an abortion are rarely seen in Nigerian health facilities [8]. Hence, studies examining the prevalence of PAC-seeking in a facility-based study would almost certainly overestimate PAC prevalence because the multitude of women who should form the denominator (women who had an abortion) of such estimation would be grossly missing. This makes a community-based survey more accurate when estimating PAC behavior.

The real potential for women to deliberately distort their abortion experience motivated this study’s investigation of behavioral intention. This approach has been used in investigating behavioral intentions in other culturally sensitive contexts. A study [18] investigated the intention of men who have sex with men to participate in voluntary counseling and HIV testing and to access free condoms in Indonesia. This study provides evidence to support two important constructs of TPB, which are (i) motivation to comply (spousal/partner’s support); and (ii) control factors (facility-related barriers). Our study ascertains that facility-related barriers are significantly associated with women’s intention to seek PAC. This finding corroborates the outcomes from earlier studies. For instance, a study [27] revealed that respecting patients' confidentiality and privacy improved their health-seeking behavior. Another study [28] also found that insufficient supplies of essential drugs and a shortage of manpower had an adverse effect on health-seeking behavior.

In this study, lack of service confidentiality and unavailability of abortion-specific equipment in facilities were the commonest perceived barriers to seeking PAC among women in Osun State. This is to be expected because confidentiality is the bedrock of trust between providers and patients upon which patient’s personal and health information are disclosed [29]. In the context of abortion in a legally restrictive setting like Nigeria’s, confidentiality becomes much more important. Women might have had a clandestine abortion because they did not want anyone sometimes their spouses/partners to know that they were pregnant in the first place. If they perceived that seeking PAC in a health facility would reveal their pregnancy status, they may jettison their intention to seek PAC in the facility and possibly seek an alternative. Even women whose abortion decision was taken jointly with their partners/spouses, would desire that outsiders are not in the know of their pregnancy termination. Moreover, in many Nigerian health facility settings, the waiting rooms are usually full [12] and Obstetrics and Gynecologic (O&G) clinics are often not demarcated from other departments. Hence, women visiting O&G for PAC are likely to be recognized by others. This potentially explains the importance placed on confidentiality in PAC services by the respondents.

It is worth noting that the Yoruba people of Osun state have culturally transited from a historical state where virginity used to be a precondition for a lady to be considered virtuous for marriage, to a state where men rarely bother themselves about the virginity status of their potential brides [30]. Instead, a lady’s abortion history has largely replaced the narrative of virginity. Today, women who have an abortion history are likely to be seen as promiscuous or perceived as potential victim of secondary infertility. This is another plausible reason why lack of confidentiality in PAC services would make women have an unhealthy intention about seeking PAC in the facility.

This study also found that the availability of spousal/partner support raises the odds of a healthy PACSI among women. This variable was adapted from the TPB’s normative factors (what significant others think about the given behavior). Again, the significant influence of these normative factors in predicting PAC intention provides evidence to support TPB. This result aligns with the findings of previous studies which show the significant influence of household support on health-seeking behavior [31, 32]. It is not unexpected that women whose spouses/partners would provide financial and moral support for PAC would have a healthy intention about PAC. However, in contrast with another study’s findings [33], this study reports that the opinion of significant others had no significant influence on women’s PACSI. This also is in contrast with a TPB’s postulation which states that normative beliefs (how significant others think of behavior) are a significant factor influencing behavioral intention. This result indicates that women could act in a way not consistent with what people around them feel. A plausible reason why women could do this is that they are the ones who know where the shoes pinch. If relatives, friends, or even religious leaders think it necessary to seek PAC, women may decline if they find it unnecessary. On the other hand, if women feel PAC would save their lives from abortion complications, they are likely to defile what significant others think and seek the needed care. This is a significant pointer to the limitation of the TPB used.

This study filled the knowledge gap created by the lack of community-based evidence on women’s intention to seek PAC when the need arises. This is despite abundant evidence that PAC prevents death and complications caused by unsafe abortion which is widespread in Nigeria. The study enabled the understanding of how perceived facility-related barriers would influence individual women’s health-seeking behavior if they have an abortion. It helped in unlocking insight into perceived barriers which would enable healthcare practitioners and policymakers to understand where barriers exist and how to encourage a positive behavioral change. The use of an electronic data kit for data collection prevented errors and this contributed to the data quality. However, inferences drawn in the study are limited by a few drawbacks. One, the study’s use of TPB suggests that complex human behavior could accurately be predicted through intentions and this may not be always correct. Second, the reported health facility-related barriers were based on the perception of the respondents and may not truly reflect the situation of PAC service features in Osun state. Lastly, women’s reports of intention to seek PAC might have been underestimated because some never experienced abortion or its complication to have been able to know the importance of PAC to their health.

## Conclusion

This study shows that approximately one-third of women in Osun state had the intention to seek PAC in a health facility should they have an abortion. Perceived lack of service confidentiality and unavailability of equipment specific to abortion were the two most commonly reported health facility-related barriers which had a significant influence on women’s PAC intention. Being supported by spouses and skillfully employed significantly increased the odds of seeking PAC in a health facility. Therefore, the management of health facilities is strongly advised to overhaul PAC service provision in a way that patient’s confidentiality will be preserved. It is recommended that future health promotion interventions focus on improving the public perception of healthcare services by re-assuring the public about service confidentiality and the availability of needed equipment in the facilities. Given that Osun state was chosen for this study because of its relatively high unwanted pregnancy rate, but which was higher in south-south region, it is recommended that PACSI be investigated among women in south-south states.

## Data Availability

Data analyzed and the Stata dofile used for the analysis are available upon request. The corresponding author should be contacted for this.

## Abbreviations

PAC: Post Abortion Care
NDHS: Nigeria Demographic and Health Survey
PACSI: Post Abortion Care-seeking Intention
HFRB: Health Facility-related Barriers
SDGs: Sustainable Development Goals
TPB: Theory of Planned Behaviour
NPC: National Population Commission
VIF: Variance Inflation Factor
OR: Odds Ratio
